# Prognostic value of nutritional risk index in esophageal cancer: A protocol for systematic review and meta-analysis

**DOI:** 10.1097/MD.0000000000046269

**Published:** 2026-05-12

**Authors:** Ze-yuan Liu, Ben Liu

**Affiliations:** a Department of Radiotherapy, Nanjing Jiangning Hospital, Nanjing, China; b Department of Oncology, Shuyang County Hospital of Traditional Chinese Medicine, Suqian, China.

**Keywords:** esophageal neoplasms, meta-analysis, nutritional risk index, prognosis, survival analysis

## Abstract

**Background:**

The nutritional risk index (NRI) and its modified version, the geriatric nutritional risk index (GNRI), are simple formula-based tools reflecting nutritional and inflammatory status. While several studies have investigated their prognostic value in esophageal cancer (EC), results remain inconsistent, and no prior meta-analysis has directly compared NRI and GNRI in this context. Therefore, this meta-analysis was conducted to thoroughly assess the prognostic significance of NRI/GNRI in EC.

**Methods:**

Possible studies were obtained from Embase, Web of Science, PubMed, and Cochrane Library from their inception to November 2024. A relative ratio (RR) and a hazard ratio (HR) were extracted and pooled to analyze the correlation between the prognosis of EC and NRI/GNRI.

**Results:**

The final meta-analysis included 10 studies, with 2600 patients. As displayed in the pooled results, high NRI/GNRI predicted superior overall survival (HR = 1.78, 95% confidence interval [CI]: 1.53–2.07, *P *< .01) and distant metastasis-free survival/progression-free survival/recurrence-free survival (HR = 1.95, 95% CI: 1.52–2.51, *P* < .01). The association was presented between low NRI/GNRI and more lymph node metastasis (RR = 1.18, 95% CI: 1.01–1.38, *P *= .040), deeper tumor invasion (RR = 1.18, 95% CI: 1.01–1.38, *P *= .040), as well as advanced tumor stage (RR = 1.32, 95% CI: 1.10–1.58, *P *= .003).

**Conclusion:**

Both NRI and GNRI are independent prognostic factors in EC. Incorporating nutritional risk screening into routine clinical practice may support early intervention and improve treatment outcomes, particularly in surgical patients.

## 1. Introduction

Esophageal cancer (EC) is the second most commonly diagnosed tumor of the digestive system after gastric cancer and ranks among the 10 most prevalent malignancies worldwide.^[1]^ Despite advancements in EC treatment, survival rates for locally advanced cases remain poor, with a 5-year survival rate of only 15% to 25%.^[2]^ Among EC patients, malnutrition is a prevalent issue, with rates as high as 90% during radiationtherapy.^[3]^ Malnutrition has severe implications, including diminished quality of life, impaired immune response, and reduced chemotherapy efficacy.^[4]^ Current European guidelines emphasize the importance of nutritional counseling and comprehensive nutritional assessments for all patients undergoing radiotherapy for gastrointestinal cancer.^[5]^

While several tools assess nutritional status, including the Physiological and Operative Severity Score for the Enumeration of Mortality and Morbidity and the Prognostic Nutritional Index, these are often deemed too complex for routine clinical use.^[6]^ Recently, the nutritional risk index (NRI) has emerged as a simpler and practical alternative. NRI integrates albumin levels and body weight to assess nutritional status, facilitating its adoption in clinical settings. A modified version, the geriatric nutritional risk index (GNRI), replaces usual body weight with ideal body weight and has been widely applied in elderly or frail cancer patients. Numerous studies have demonstrated the prognostic value of NRI and GNRI in digestive cancers, including EC.^[7,8]^ Despite its potential, the role of NRI in predicting EC prognosis is not without controversy. For instance, some studies report a strong association between low NRI and poor survival outcomes, whereas others highlight that even nutritional interventions fail to significantly improve outcomes in patients with aggressive cancers.^[9,10]^ Furthermore, differences in NRI calculation methods – such as using actual versus ideal body weight – have led to inconsistencies in defining malnutrition thresholds.^[11]^ Additionally, while studies indicate a correlation between low NRI and postoperative complications, the exact relationship remains poorly understood, particularly concerning respiratory outcomes.

In recent years, significant progress has been made in understanding the prognostic value of NRI, as evidenced by several landmark studies. For instance, Oh et al^[12]^ uncovered that pretreatment NRI risk category could predict overall survival (OS) in head and neck cancer. In a meta-analysis published by Yu et al,^[10]^ lower GNRI was significantly associated with poorer OS and progression-free survival (PFS) in EC. Furthermore, Shi et al (2023) identified GNRI as an independent predictor of mortality in lung cancer patients with advanced heart failure, expanding its clinical relevance.^[8]^ Similarly, Zhou et al^[13]^ found that low GNRI was significantly related to worse OS and cancer-specific survival. Yang et al^[14]^ and Fan et al^[15]^ further supported these findings, highlighting GNRI as an important prognostic tool for survival outcomes in EC patients. NRI was initially introduced by Buzby et al^[16]^ and later modified by Bouillanne et al,^[17]^ resulting in the GNRI. Despite slight differences in the formulas, the relevant standards for malnutrition assessment have been adjusted accordingly. As reported by Yamada et al,^[11]^ a comparison of the NRI values calculated by the 2 formulas revealed minimal differences. Therefore, while these studies have established the prognostic value of GNRI, this meta-analysis was the first to systematically evaluate both NRI and GNRI in EC. This broader focus allows for a more comprehensive assessment of the prognostic value of nutritional indices for EC, incorporating both NRI and GNRI.

This study retrieved available articles for a meta-analysis to thoroughly analyze the prognostic value of NRI/GNRI in EC and investigate relevant clinicopathological factors.

## 2. Material and methods

The reports of systemic review and meta-analysis were based on the Strengthening the Reporting of Observational Studies in Epidemiology guidelines^[18]^ and the Preferred Reporting Items for Systematic Reviews and Meta-Analyses guidelines.^[19]^ Moreover, this meta-analysis was registered in PROSPERO (registration number: CRD42024608919).

As this study is a systematic review and meta-analysis of previously published literature, no new human participants were enrolled and no individual patient data were collected. Therefore, approval by an institutional review board or ethics committee and informed consent were not required.

### 2.1. Search strategy

The search aimed to retrieve all literature related to NRI/GNRI with EC. The literature was compiled from the Embase, Cochrane Library, PubMed, and Web of Science databases from their inception to April 2020. The enrolled keywords were “Esophageal Neoplasms” or “EC,” “nutritional risk index” or “NRI,” “geriatric nutritional risk index” or “GNRI,” “nutrition assessment,” and “prognosis.” The retrieval and usability evaluations of the integrated databases were conducted independently by 2 reviewers. In case of discrepancies, the final judgment was made by a third assessor. Moreover, after the comprehensive search, a second manual search was conducted on the collected documents.

### 2.2. Study selection

The inclusion criteria were as displayed: studies regarding the prognostic value of NRI or GNRI in EC; studies that reported associations between NRI/GNRI and either survival outcomes or clinicopathological characteristics; studies with sufficient data to count 95% confidence interval (CI) and hazard ratio (HR); and only studies with a sample size ≥30 included to avoid publication bias.

NRI and GNRI are generally calculated as follows: NRI = 1.519 × albumin (g/L) + (41.7 × present/usual weight); GNRI = 1.489 × albumin (g/L) + (41.7 × present/ideal weight). Given their close methodological relationship and similar prognostic implications, both NRI and GNRI studies were included in this analysis, and subgroup analyses were conducted to compare their prognostic performance separately.

### 2.3. Data collection and quality assessment

Data extracted from each enrolled study included author, title, country, publication year, treatment regimen, sample size, univariate or multivariate analysis, follow-up time, pathological type, tumor stage, survival outcome, HR, and 95% CI. Preference was given to multivariate analysis when both multivariate and univariate analysis results were available, as only multivariate analysis controls for confounding factors.

Quality assessment was carried out using the Newcastle-Ottawa Scale.^[20]^ Studies were scored on a scale of 0 to 9, where studies scoring ≥6 were considered high quality. Quality assessment and data extraction were independently conducted by 2 researchers.

### 2.4. Statistical analysis

The prognostic value of NRI/GNRI in EC was evaluated using 95% CI and HR. In all studies, HR was defined as low NRI/GNRI versus high NRI/GNRI. The reciprocal of 95% CI and HR was taken in studies where HR was high NRI versus low NRI. Precise HR values were not reported by all studies, but Kaplan–Meier survival curves were provided. In that case, data were extracted from survival curves using Engauge Digitizer version 11.1, and HR was then calculated using the spreadsheet provided by Tierney et al.^[21]^ Notably, HR < 1 indicated that a low NRI/GNRI was linked to a reduced risk of death, whereas HR > 1 suggested that a low NRI/GNRI elevated the risk of death. It was deemed to be not significant if the 95% CI of HR included the value of 1. Assessment was also conducted on related clinicopathological factors potentially influencing NRI/GNRI in EC with 95% CI and a relative risk (RR).

Heterogeneity among studies was measured using *I*^2^ statistics.^[22]^ If no significant heterogeneity was detected (*I*^2^ < 50%), a fixed-effects model was applied to pool data. Otherwise, a random effect model was employed. Sensitivity and subgroup analyses were performed to identify sources of heterogeneity. Publication bias was evaluated using Begg’s and Egger’s tests,^[23,24]^ with *P* < .05 indicating significant publication bias. The trim-and-fill method was applied to adjust HR. If publication bias was presented among studies. *P* < .05 represented the statistical significance threshold. All statistical analyses were conducted using Stata Statistical software, version 15.0 (Stata Corporation, College Station).

## 3. Results

### 3.1. Study selection

**Figure 1.**
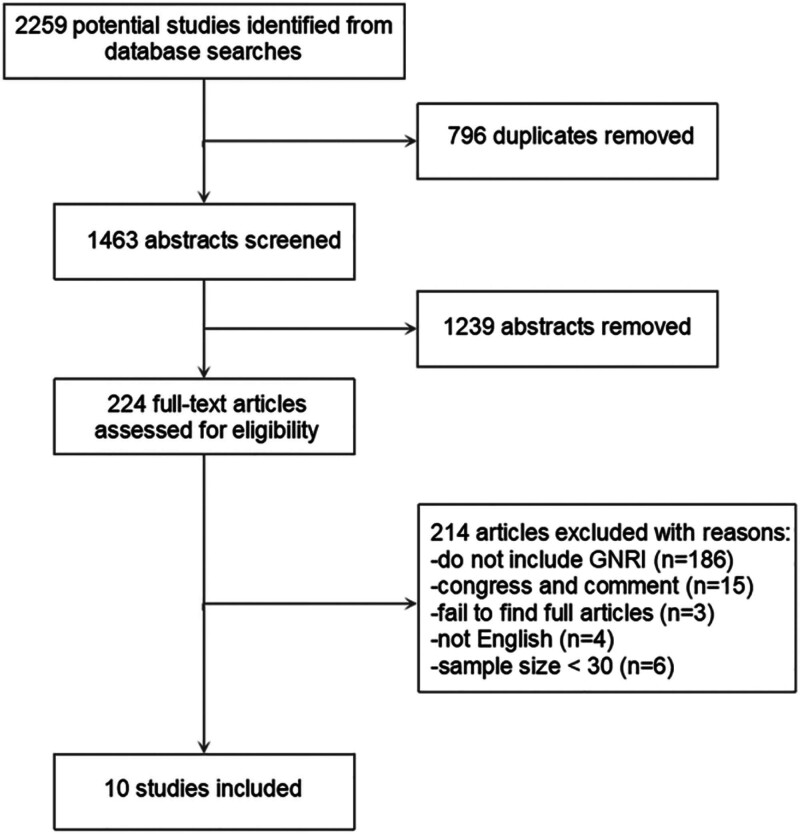
Flow chart of the study selection process. GNRI = geriatric nutritional risk index.

As shown in Figure 1, a total of 2259 associated studies were collected. After excluding duplicate articles (n = 796), 1239 were excluded based on abstract screening. Following the full text in sequence, studies were further excluded for the following reasons: lack of NRI index (n = 186), non-English language articles (n = 4), congress (n = 9), comment (n = 6), total sample size less than 30 (n = 6), and inaccessible full text (n = 3). Ultimately, 10 studies^[9,25–33]^ meeting the inclusion criteria were obtained.

A total of 1600 patients were included in the meta-analysis, with a median sample size of 146 (range: 44–258). The literature was published from 2014 to 2019 in China,^[26–28]^ Japan,^[25,29–32]^ the United Kingdom,^[9]^ and France.^[33]^ Among these studies, six studies specifically analyzed esophageal squamous cell carcinoma,^[26–31]^ while the pathological types of esophageal carcinoma were not specified in the others.^[9,25,32,33]^ Besides, six focused on the nutritional status of the patients undergoing chemoradiotherapy,^[9,26–29]^ three on those receiving surgery or neoadjuvant therapy followed by surgery,^[30–32]^ and one on patients undergoing only surgery.^[25]^ Additionally, only one trial conducted the multicenter retrospective study^[9]^ while the rest conducted the single-institution retrospective study.^[25–33]^ The quality assessment scores of the included studies ranged from 5 to 7 based on the Newcastle-Ottawa Scale. The basic characteristics of the included literature were displayed in Table 1.

**Table 1:** The information of included studies and quality.

Author	Country	Centers	Study design	Sample sizes	Tumor type	Ages median/mean (range)	Clinical stage	Treatment regimen	NRI/GNRI	Mean value	Cutoff value	Median follow-up period (mo)	Survival analysis	Outcome	Quality scores
Yamana et al^[25]^	Japan	Single center	Retrospective	122	Mixed	63.9 ± 9.1 (43–83)	04/Ⅰ26/Ⅱ32/Ⅲ47/Ⅳ13	Surgery	GNRI	96.2 ± 10.1	90	NR	Multivariate analysis	OS	6
Wang et al^[26]^	China	Single center	Retrospective	52	ESCC	74 (70–83)	Ⅰ-Ⅱ20/Ⅲ-Ⅳ32	CRT/RT	GNRI	NR	92, 98	NR	Multivariate analysis/univariate analysis	OS/PFS/ORR	5
Zhou et al^[27]^	China	Single center	Retrospective	149	ESCC	79 (75–91)	Ⅰ-Ⅱ64/Ⅲ85	Definitive CRT/RT	NRI	NR	100	22.5	Multivariate analysis	OS/DMFS	6
Bo et al^[28]^	China	Single center	Retrospective	239	ESCC	67.9 ± 5.9 (60–88)	Ⅰ22/Ⅱ53/Ⅲ26/Ⅳ71	Radiotherapy	GNRI	NR	92, 98	40.4	Multivariate analysis	OS	7
Sakanaka et al^[29]^	Japan	single center	Retrospective	44	ESCC	66 (55–84)	Ⅰ44	CRT	NRI	NR	100	72	NA	OS	5
Kubo et al^[30]^	Japan	Single center	Retrospective	240	ESCC	63.4 (NR)	Ⅰ70/Ⅱ51/Ⅲ105/Ⅳ14	Surgery/NCRT + surgery	GNRI	97.4 ± 6.89	92	NR	Multivariate analysis	OS/CSS	6
Migita et al^[31]^	Japan	Single center	Retrospective	137	ESCC	NR	Ⅰ48/Ⅱ33/Ⅲ56	Surgery/NCRT + surgery	GNRI	99.9 ± 7.8	98	NR	Multivariate analysis/univariate analysis	OS/RFS	7
Yamana et al^[32]^	Japan	Single center	Retrospective	216	Mixed	NR	NR	Surgery/NCRT + surgery	GNRI	96.1 ± 9.72	92	NR	Multivariate analysis/univariate analysis	OS	7
Cox et al^[9]^	England	Multicentre	Random	258	Mixed	66.8 (NR)	Ⅰ8/Ⅱ72/Ⅲ23/Ⅳ155	CRT	NRI	NR	100	25	Multivariate analysis/univariate analysis	OS	7
Clavier et al^[33]^	France	Single center	Retrospective	143	Mixed	65 (42–81)	Ⅰ-ⅡA60/ⅡB-ⅣA83	CRT/RT	NRI	NR	97.5	20.8	Multivariate analysis	OS/RFS	6

CRT = chemoradiotherapy, CSS = cancer-specific survival, DMFS = distant metastasis-free survival, ESCC = esophageal squamous cell carcinoma, GNRI **=** geriatric nutritional risk index, NCRT = neoadjuvant chemoradiotherapy, NR = not report, NRI **=** nutritional risk index, ORR = objective response rate, OS = overall survival, PFS = progression-free survival, RFS = recurrence-free survival, RT = radiotherapy.

### 3.2. Primary finding

**Figure 2.**
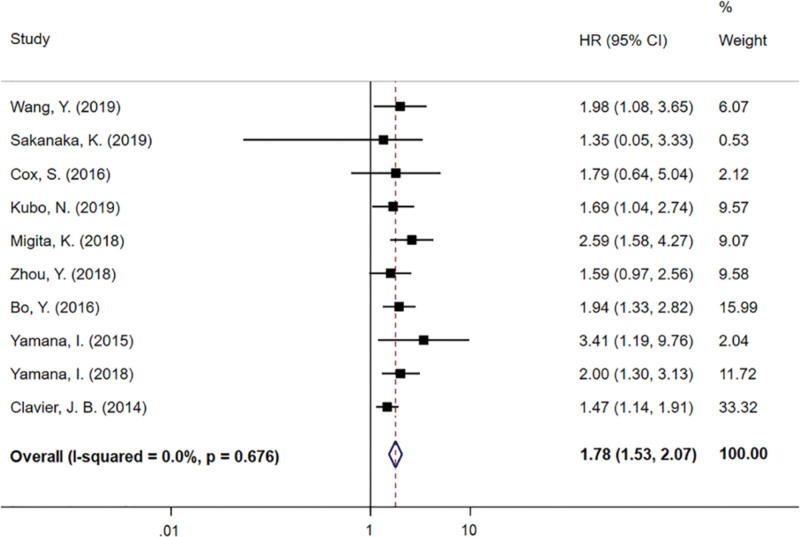
Meta-analyses of the associations between nutritional risk index and overall survival. 95% CI = 95% confidence interval, HR = hazard ratio.

The association between NRI/GNRI and OS was reported in 10 trials, of which 6 specifically analyzed GNRI. Since the heterogeneity test (*I*^2^ = 0.0%, *P *= .676) did not reveal significant heterogeneity, a fixed-effects model was selected to merge HR as well as 95% CI. The meta-analysis indicated a significant association between high NRI/GNRI and prolonged OS, with a pooled HR of 1.78 (95% CI: 1.53–2.07, *P* < .01) (Fig. 2).

**Figure 3.**
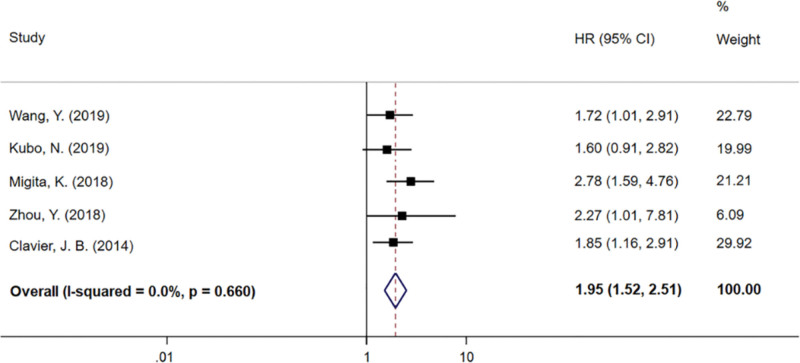
Meta-analyses of the associations between nutritional risk index and DMFS/PFS/RFS. 95% CI = 95% confidence interval, DMFS = distant metastasis-free survival, HR = hazard ratio, PFS = progression-free survival, RFS = recurrence-free survival.

There were 5 studies reporting the relationship between NRI/GNRI and distant metastasis-free survival (DMFS)/PFS/recurrence-free survival (RFS). A fixed-effects model was also selected, as no significant heterogeneity (*I*^2^ = 0.0%, *P* = .660) was detected in the heterogeneity test. The results revealed that high NRI/GNRI was associated with longer DMFS/PFS/RFS outcomes, with an HR of 1.95 (95% CI: 1.52–2.51, *P* < .01) (Fig. 3). This further supported the role of NRI/GNRI as an important predictor of survival, beyond just OS, indicating a consistent trend across different survival metrics.

### 3.3. Subgroup analysis

Despite the low heterogeneity, a subgroup analysis was conducted in this meta-analysis based on patient characteristics (areas, sample sizes, NRI/GNRI, type of cancers, cutoff values) and therapeutic methods to better determine the relationship between NRI/GNRI and patient prognosis (Table 2). No significant heterogeneity was observed among subgroups. Both NRI and GNRI consistently showed prognostic value, with lower scores predicting poorer OS. However, the pooled HR indicated that GNRI (HR = 2.05, 95% CI: 1.67–2.51) demonstrated a stronger association with OS than NRI (HR = 1.51, 95% CI: 1.21–1.88). This difference suggests that GNRI, which incorporates ideal weight in place of usual weight, may capture nutritional risk more sensitively, particularly in elderly or frail patients. When stratified by treatment modality, the prognostic effect of nutritional indices appeared more pronounced in surgical patients (HR = 2.12, 95% CI: 1.63–2.7) than in non-surgical patients (HR = 1.64, 95% CI: 1.37–1.97), most of whom received definitive chemoradiotherapy or radiotherapy.

**Table 2:** Results of subgroup analysis of pooled hazard ratio of overall survival.

Subgroup analysis	No. of studies	No. of patients	Pooled HR and its 95% CI	Meta-regression (*P* value)	Heterogeneity	
					*I*^2^ (%)	*P* value
Region						
China	3	440	1.83 (1.40–2.39)	<.001	0.0	.786
Japan	5	759	2.10 (1.616–2.73)	<.001	0.0	.643
European countries	2	401	1.49 (1.16–1.92)	.002	0.0	.718
NRI/GNRI						
NRI	4	594	1.51 (1.21–1.88)	<.001	0.0	.979
GNRI	6	1006	2.05 (1.67–2.51)	<.001	0.0	.779
Sample size						
<200	6	647	1.72 (1.42–2.08)	<.001	16.4	.308
≥200	4	953	1.88 (1.48–2.39)	<.001	0.0	.960
Cutoff value						
<100	7	1149	1.81 (1.54–2.12)	<.001	5	.389
≥100	3	451	1.61 (1.05–2.47)	.030	0.0	.965
Type of cancer						
ESCC	6	861	1.91 (1.55–2.36)	<.001	0.0	.794
Mixed	4	739	1.65 (1.33–2.05)	<.001	10.1	.342
Surgical condition						
Surgery	4	715	2.12 (1.63–2.76)	<.001	0.0	.868
No surgery	6	885	1.64 (1.37–1.97)	<.001	0.0	.507

95% CI = 95% confidence interval, ESCC = esophageal squamous cell carcinoma, GNRI = geriatric nutritional risk index, HR = hazard ratio, NRI = nutritional risk index.

### 3.4. NRI/GNRI and clinicopathological features

The relationship between NRI/GNRI and key clinicopathological features was displayed in Table 3. High NRI/GNRI was associated with the earlier tumor stage (RR = 1.32, 95% CI: 1.10–1.58, *P* = .003). As opposed to patients without lymph node metastasis, patients with lymph node metastasis exhibited a reduced NRI/GNRI (RR = 1.18, 95% CI: 1.01–1.38, *P* = .040) and shallower tumor infiltration (RR = 1.51, 95% CI: 1.18–1.95, *P* = .001). However, no association was observed between NRI/GNRI and gender or tumor locations.

**Table 3:** Results of meta-analysis of NRI/GNRI and clinicopathological features.

Clinicopathological feature	No. of studies	No. of patients	Pooled RR and its 95% CI	Meta-regression (*P* value)	Heterogeneity	
					*I*^2^ (%)	*P* value
Gender (male vs female)	5	887	0.99 (0.90–1.08)	.781	27.0	.242
Tumor stage (Ⅰ/Ⅱ vs Ⅲ/Ⅳ)	4	750	1.32 (1.10–1.58)	.003	45.8	.136
T stage (T1–2 vs T3–4)	3	536	1.51 (1.18–1.95)	.001	0.0	.512
N stage (N0 vs N1)	4	775	1.18 (1.01–1.38)	.040	22.0	.279
Tumor location (cervical/upper vs middle/lower)	3	510	1.09 (0.84–1.40)	.514	0.0	.478

95% CI = 95% confidence interval, GNRI = geriatric nutritional risk index, NRI = nutritional index, RR = relative ratio.

### 3.5. NRI/GNRI and postoperative complications

The relationship between NRI/GNRI and postoperative complications was also examined in this meta-analysis (Table 4). Although there was no significant correlation between NRI/GNRI and overall complications, a subset analysis found that lower NRI/GNRI was associated with a higher incidence of respiratory complications in 2 of 3 studies (Yamana et al and Kubo et al).^[25,30]^ This result suggested that NRI/GNRI might be particularly beneficial for assessing respiratory risk in surgical patients, likely due to its link with immune and muscle function.

**Table 4:** Results of meta-analysis of NRI/GNRI and complications.

Complication	No. of studies	No. of patients	Pooled RR and its 95% CI	Meta-regression (*P* value)	Heterogeneity	
					*I*^2^ (%)	*P* value
Respiratory disease (no vs yes)	3	499	1.29 (0.89–1.86)	.178	85.6	.001
Cardiovascular disease (no vs yes)	2	259	0.89 (0.24–1.64)	.503	80.0	.026
Diabetes mellitus (no vs yes)	3	498	1.01 (0.94–1.08)	.784	0.0	.466
Liver disease (no vs yes)	2	259	0.95 (0.90–1.01)	.078	0.0	.516

95% CI = 95% confidence interval, GNRI = geriatric nutritional risk index, NRI = nutritional index, RR = relative ratio.

### 3.6. Sensitivity analysis and publication bias

**Figure 4.**
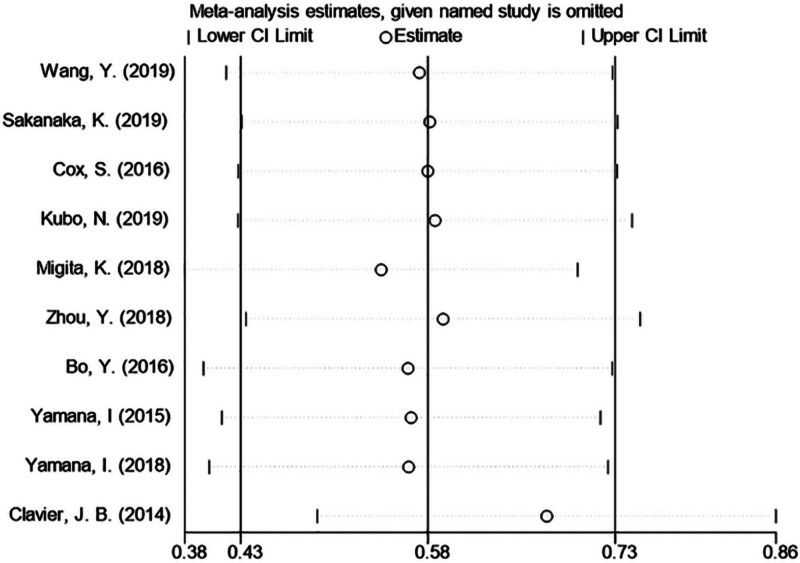
Sensitivity analysis of the relationship between nutritional risk index and overall survival.

**Figure 5.**
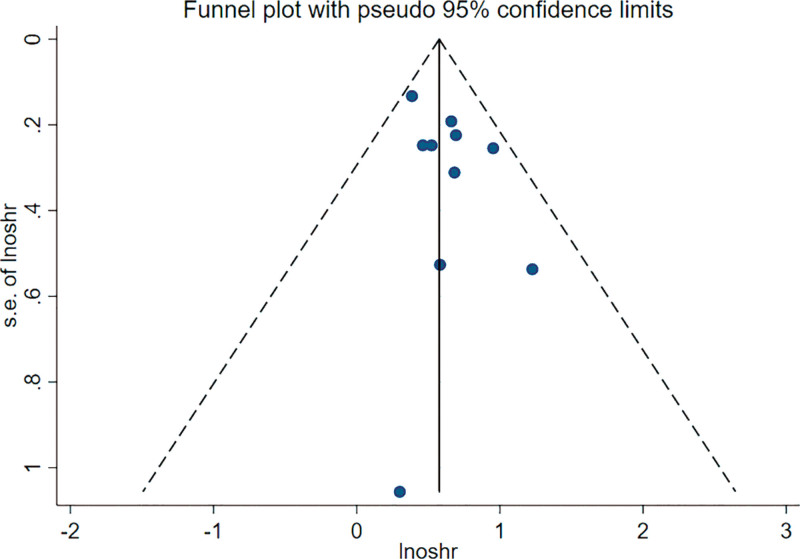
Funnel plot of publication bias for nutritional risk index and overall survival.

The sensitivity analysis (Fig. 4) demonstrated that the merged results were not significantly affected by removing HR from any study, aligning with the subgroup analysis findings. Begg’s test, Egger’s test, and the funnel plot were utilized to evaluate the publication bias of this meta-analysis (Fig. 5). No research deviated from the precision line. Both Egger’s test (*P* = .182) and Begg’s test (*P* = .531) indicated the absence of publication bias in this meta-analysis.

## 4. Discussion

This meta-analysis demonstrated that the NRI/GNRI was a significant prognostic indicator for patients with EC, showing a positive correlation with OS, PFS, DMFS, and RFS. Besides, this study underscored the relevance of nutritional assessment as an essential component of EC prognosis by consistently linking high NRI/GNRI with improved outcomes on these metrics. Clinically, these results suggested that evaluating NRI/GNRI could provide a straightforward and effective method for stratifying patients based on survival risk, enabling more personalized management strategies. Given the high mortality and complication rates in EC, incorporating NRI/GNRI into routine assessments holds promise for improving patient outcomes through proactive, nutrition-focused support.

The current analysis demonstrated that high NRI/GNRI reflects superior nutritional status, which is closely linked to better systemic immune function and reduced systemic inflammation.^[34,35]^ Malnutrition is known to impair immune responses by reducing T-cell activity and lymphocyte count, thereby weakening the body’s ability to combat tumor progression.^[36,37]^ Moreover, albumin, a key component of the NRI/GNRI formula, is an established marker of both nutritional status and systemic inflammation.^[7]^ Low albumin levels are associated with increased inflammatory cytokines, which are implicated in tumor growth and metastasis.^[38,39]^ Therefore, patients with higher NRI/GNRI scores likely benefit from enhanced immune surveillance and reduced inflammatory tumor microenvironments, contributing to improved survival outcomes.

The observed association between low NRI/GNRI and higher rates of respiratory complications may stem from the role of malnutrition in muscle function in this study. Studies suggest that poor nutritional status contributes to respiratory muscle weakness, leading to a higher likelihood of postoperative pulmonary complications.^[25,37,40]^ Furthermore, systemic inflammation exacerbated by malnutrition could impair wound healing and increase susceptibility to infections, further complicating recovery.^[41,42]^ These findings underscore the importance of preoperative nutritional optimization, particularly for high-risk patients undergoing surgery for EC.

Subgroup analysis revealed stronger prognostic value of NRI/GNRI in patients undergoing surgical treatment compared to those receiving chemoradiotherapy. This difference may arise because surgical patients often experience significant physiological stress, which amplifies the impact of baseline nutritional status on recovery and survival outcomes.^[43]^ Conversely, in non-surgical patients, the aggressive progression of the disease and treatment-related toxicities might overshadow the influence of nutrition.^[44]^ Nevertheless, nutritional status is also known to influence treatment tolerance, immune competence, and toxicity management during chemoradiotherapy, and thus it is plausible that NRI/GNRI could provide prognostic information in these settings as well. However, the available data were insufficient to allow modality-specific subgroup analyses beyond surgery versus non-surgery in this study. Future prospective studies are warranted to clarify the prognostic utility of NRI/GNRI in patients undergoing chemotherapy and radiotherapy.

The association between NRI/GNRI and earlier tumor stages suggests that patients with better nutritional status may experience slower tumor progression. The inflammatory-nutritional-immune provides a plausible explanation: malnutrition and systemic inflammation are known to promote angiogenesis and tumor invasiveness, leading to more advanced disease.^[45,46]^ Thus, high NRI/GNRI may serve as a surrogate marker for a less aggressive tumor microenvironment, offering a potential therapeutic window for intervention.

Furthermore, the prognostic utility of NRI/GNRI across multiple survival metrics (OS, PFS, DMFS, and RFS) underscored the need for a multidisciplinary approach to cancer care. This included collaboration among oncologists, surgeons, and nutritionists to monitor and manage the nutritional status of patients with EC. Given the current findings, incorporating NRI/GNRI assessment into routine pretreatment evaluations could facilitate more tailored treatment plans, potentially improving quality of life and survival outcomes.

The findings of this study have significant clinical implications. Incorporating NRI/GNRI assessments into routine pretreatment evaluations can provide a cost-effective tool for risk stratification. For surgical patients, preoperative nutritional optimization programs may reduce complications and improve recovery. For non-surgical patients, early identification of malnutrition allows for timely interventions to mitigate treatment-related toxicities and support immune function.

Importantly, while several prior meta-analyses have evaluated the prognostic value of GNRI in esophageal cancer,^[10,13,47]^ our study was the first to both NRI and GNRI within a single analysis and to directly compare their prognostic performance. In addition, we extended the analysis beyond overall survival to include multiple endpoints and further investigated clinicopathological associations and postoperative complications. These aspects distinguish our work from previous studies and provide a more comprehensive understanding of the prognostic role of nutritional indices in EC.

However, there are some limitations in this meta-analysis. First, the number of eligible studies was relatively small, which may restrict the generalizability of our findings across different populations and clinical settings. Larger multicenter investigations are needed to strengthen the external validity of the results. Second, all included studies are retrospective in nature, which inherently introduces risks of selection bias, incomplete data collection, and residual confounding, thereby reducing the robustness of causal inference. Although multivariate-adjusted estimates were preferentially extracted to minimize these biases, prospective studies will be required to confirm our conclusions. Third, the majority of included studies were conducted in East Asia (China and Japan). Genetic background, dietary patterns, and healthcare practices specific to these populations may influence the prognostic value of NRI/GNRI, which limits the applicability of our findings to non-Asian cohorts. Fourth, HR and 95% CI were not provided directly in one study; instead, they were obtained using the method recommended by Tierney et al^[21]^; however, some errors may occur compared with the true value. In addition, the tumor types included in some studies were not identified, and the staging definitions varied, potentially affecting the results. Finally, the ideal weight in some studies was calculated using the body mass index of 22, while others adopted the original NRI equation or the Lorentz formula^[17]^; although prior evidence indicates minimal difference between the two, these methodological inconsistencies may have influenced the results.^[11]^

## 5. Conclusion

In summary, this meta-analysis confirms that the nutritional risk index is a significant prognostic tool for EC patients, strongly correlating with improved survival outcomes and reduced postoperative complications. High NRI/GNRI reflects better nutritional and immune status, which mitigates systemic inflammation and enhances recovery, particularly in surgical patients. Incorporating NRI/GNRI into routine clinical assessments can enable early identification of high-risk patients and guide tailored nutritional interventions. However, further prospective studies and standardized evaluation methods are needed to validate these findings and optimize the clinical application of NRI/GNRI in EC management.

## Author contributions

**Conceptualization**: Ze-yuan Liu, Ben Liu.

**Data curation**: Ze-yuan Liu, Ben Liu.

**Formal analysis**: Ze-yuan Liu.

**Methodology**: Ze-yuan Liu.

**Resources**: Ben Liu.

**Software**: Ben Liu.

**Supervision**: Ze-yuan Liu, Ben Liu.

**Writing – original draft**: Ze-yuan Liu.

**Writing – review & editing**: Ben Liu.
